# Effect of dietary intervention on the prevalence of asymptomatic malaria among 6–18-month-old children in rural Malawi

**DOI:** 10.1186/s12936-023-04701-4

**Published:** 2023-09-11

**Authors:** Hany Sady, David Chaima, Lotta Hallamaa, Emma Kortekangas, Ulla Ashorn, Jomo Banda, Charles Mangani, Kenneth Maleta, Per Ashorn, Yue-Mei Fan

**Affiliations:** 1 Center for Child, Adolescent and Maternal Health Research, Faculty of Medicine and Health Technology, Tampere University, Arvo Ylpön Katu 34, 33014 Tampere, Finland; 2 Faculty of Medicine and Health Sciences, Hodeidah University, Hodeidah, Yemen; 3 Kamuzu University of Health Sciences, Blantyre, Malawi; 4 Department of Paediatrics, Tampere University Hospital, Tampere, Finland

**Keywords:** Asymptomatic malaria, Children, Dietary Intervention, Malawi, Prevalence rate

## Abstract

**Background:**

The complex interaction between malaria and undernutrition leads to increased mortality and morbidity rate among young children in malaria-endemic regions. Results from previous interventions suggest that improving nutritional status of young children may reduce the burden of malaria. This study tested a hypothesis that provision of lipid-based nutrient supplements (LNS) or corn-soy blend (CSB) supplementation to 6–18-month-old children in Malawi would reduce the prevalence of asymptomatic malaria among them.

**Methods:**

A total of 840 6-month-old children were enrolled in a randomized trial. The participants received 12-month supplementation with three different daily dietary supplementations: CSB, soy-LNS, or milk-LNS, and one control group without supplementation. The prevalence rate of asymptomatic *Plasmodium falciparum* was determined by real-time PCR from the participant’s dried blood spots (DBS) collected at the baseline and every 3 months. The global null hypothesis was tested using modified Poisson regression to estimate the prevalence ratio (PR) between the control group and three intervention groups at all ages combined. All the models were adjusted for malaria at baseline, season of DBS sample collection, site of enrolment, and household asset Z-score.

**Results:**

All children combined, the prevalence of *P. falciparum* was 14.1% at enrollment, 8.7% at 9 months, 11.2% at 12 months, 13.0% at 15 months and 22.4% at 18 months of age. Among all samples that were taken after enrolment, the prevalence was 12.1% in control group, 12.2% in milk-LNS, 14.0% in soy-LNS, and 17.2% in CSB group. Compared to children in the control group the prevalence ratio of positive malaria tests was 1.19 (95% CI 0.81–1.74; P = 0.372) in the milk-LNS group, 1.32 (95% CI 0.88–1.96; P = 0.177) in the soy-LNS group and 1.72 (95% CI 1.19–2.49; P = 0.004) in the CSB group.

**Conclusion:**

The study findings do not support a hypothesis that LNS or CSB supplementation would reduce the prevalence of asymptomatic malaria among Malawian children. In contrast, there was a signal of a possible increase in malaria prevalence among children supplemented with CSB.

**Supplementary Information:**

The online version contains supplementary material available at 10.1186/s12936-023-04701-4.

## Background

Malaria is a vector-borne and life-threatening disease estimated to be globally responsible for about 241 million cases and 627 thousand deaths per year [1]. More than 95% of the world’s malaria cases and deaths occur in Africa, and children less than 5 years constitute nearly 80% of deaths [1]. Malaria continues to be a major public health problem in Malawi with 4.4 million infected cases reported in 2018 and 98% of those were caused by *Plasmodium falciparum* [1]. Malawi represents 2% of global morbidity [1] and the highest rate of cases occurs in young children [2].

Asymptomatic malaria infections are poorly understood or have been neglected in research studies and malaria control policies. This insufficient attention may be due to lower parasitaemia intensity without symptoms which makes their detection difficult by microscopy and rapid diagnostic tests (RDTs) [3–5]. In malaria-endemic regions, the majority of malaria infections are asymptomatic [6] particularly among adults and school-age children because of acquired antimalaria immunity [7]. The persistence of asymptomatic malaria-infected carriers in the population is known to stimulate gametocytogenesis [8], facilitate the recurrence of malaria infection and serve as reservoirs and foci for sustained malaria infections in children, which interrupts malaria elimination [9–12]. This in turn, affects younger children's health with chronic anaemia and impaired growth that may reduce school performance in later life [12] and lead to undernutrition which weakens adaptive and innate immunity against parasitic infections and other infectious diseases [13]. Malaria and undernutrition are two leading causes of increased morbidity and mortality rate among children less than 5 years. This complex association persists in an interwoven vicious cycle that coexists in sub-Saharan Africa [14]. The interaction association between undernutrition and reducing asymptomatic malaria endemicity remains inconclusive with inconsistent findings [15–19].

Global health programs recommended micronutrient supplements to vulnerable children under-five years that contain various components of essential vitamins and minerals required for children’s growth to reduce malaria mortality and morbidity [20–22]. Nutrient-based interventions may reduce the severity of diseases, accelerate recovery, and stop recurrent infection by improving children’s growth which has a positive effect on the regulation and integrity of the immunity system [23, 24]. Previous studies showed a beneficial effect of lipid-based nutrient supplements (LNS) on the linear growth improvement of infants and young children (6–12 months) [25–28]. Furthermore, corn-soy blend supplements (CSB) are another common inexpensive composition and the most acceptable and obtainable components used for children with malnutrition in many low-income regions [29, 30].

LNS and CSB dietary interventions display substantial similarity in their nutritional components, featuring slight differentiations in their content. These interventions collectively contribute to the enhancement of children’s linear growth, consequently strengthening the potency of their immune response in the pursuit of disease mitigation. Limited evidence of randomized trials has found a potential protective impact of dietary interventions on the occurrence of malaria in children [17, 31]. Studies suggested that vitamin A and zinc nutritional supplements reduced malaria morbidity in young children [32, 33]. Iron supplements were found to increase the rate of malaria parasitaemia among children [34]. Essential fatty acids derivatives in the lipid base of LNS showed a significant reduction of *P. falciparum* parasitaemia in an in vitro study [35]. These contradictory impacts of dietary interventions on malaria prevalence left the topic inconclusive. The aim of this study was to test the effect of two LNS and CSB on asymptomatic *P. falciparum* malaria prevalence among young children in Malawi. The study hypothesis was that the provision of any of the three dietary interventions used for one year would reduce the prevalence rate of asymptomatic *P. falciparum* among Malawian children (6 to 18 months).

## Methods

### Study design and participants

This study is a secondary analysis of data and biological sample (dried blood spot, DBS) from a clinical trial known as Lungwena Child Nutrition Intervention 5 (LCNI-5). The trial was conducted at and around Lungwena Health Centre and Malindi Hospital catchment area, Mangochi District, Southern Malawi from 2008 to 2009. The LCNI-5 protocol and primary outcome results have been described elsewhere in detail [36]. In brief, a total of 840 6-month-old healthy infants were enrolled and randomized to receive one of three-dietary interventions or no supplements between 6 and 18 months of age: (1) control group without supplements; (2) dietary supplementation with 72 g/day fortified maize-soy flour; (3) dietary supplementation with 54 g/day of milk containing LNS; (4) dietary supplementation with 54 g/day of soy containing LNS (Table 1). Participants were visited every 2 weeks at their homes between 6 and 18 months of age and were provided with a 14-day supply of dietary supplementation.

**Table 1:** Contents of nutrients supplements

Nutrients Composition	CSB	Milk-LNS	Soy-LNS
Nutrient (g)	72	54	54
Energy (kcal)	284.4	284.8	276.1
Protein (g)	10.44	8.2	7.5
Fat (g)	3.08	17.9	18.5
Retinol (µg RE)	139	400	400
Folate (µg)	43.2	160	160
Niacin (mg)	3.456	6	6
Panthothenic acid (mg)	–	2	2
Riboflavin (mg)	0.322	0.5	0.5
Thiamin (mg)	0.128	0.5	0.5
Vit. B6 (mg)	0.336	0.5	0.5
Vit. B12 (µg)	0.86	0.9	0.9
Vit. C (mg)	48	30	30
Vit. D (µg)	–	5	5
Ca (mg)	72	366	366
Cu (mg)	–	0.4	0.4
I (µg)	–	90	90
Fe (mg)	5.46	6	6
Mg (mg)	–	78.5	78.5
Se (µg)	–	20	20
Zn (mg)	3.6	6.0	6.0
Phosphorus (mg)	–	185.6	185.6
K (mg)	–	318.6	307.3
Manganeze (mg)	–	0.60	0.60

CSB, corn-soy blend supplement; milk-LNS, milk powder containing lipid-based nutrient supplement; soy-LNS, soy-flour-containing lipid-based nutrient supplement

The trial was performed according to Good Clinical Practice guidelines and ethical standards of the Declaration of Helsinki. The protocol was approved by the College of Medicine Research and Ethics Committee, Malawi, and the Ethical Committee of Pirkanmaa Hospital District, Finland. Only participants whose guardians signed informed consent forms were enrolled in the study. This trial has been registered at www.clinicaltrials.gov (identifier NCT00524446).

### Sample collection, DNA extraction, and real-time PCR

At 3-month intervals from enrolment until 18 months old, participants were seen at the health center where they underwent a clinical check-up and blood sampling. From the blood samples taken at the clinic, 100 μl (2 spots, each 50 μl) was applied to Whatman FTA filter paper (Whatman plc, Maidstone, UK), air-dried, and placed in individually sealed plastic bags with a desiccant. The sample bags were then stored in dry condition at room temperature for about 5 years before the analyses. In total, there were 2799 DBS samples stored from this trial in 2008.

The DNA extraction of *P. falciparum* was carried out according to a slightly modified Chelex-saponin procedure that was previously published [37]. In brief, 6mm of blood spots were punched from each DBS card into a 96-deep-well plate. One ml of 1 × phosphate-buffered saline (PBS) and 50 µl of 10% saponin were added to each well containing DBS. The plate was then covered with a foil plate cover, vortexed for 20–30 s, and left to incubate overnight at 4 °C. After centrifuging and washing twice with 1 ml of PBS, 100 µl of sterile water and 50 µl of 20% Chelex 100-solution were added to each well. The plates were then incubated at 98 °C for 12 min (vortexed every 2–3 min) and thereafter centrifuged at 1500 rpm for 5 min. After that, the supernatant from each well was transferred to a new 96-deep-well plate centrifuged for 10 min at 1500 rpm, covered with foil, labelled, and stored at − 20 °C.

Quantitative polymerase chain reaction (qPCR) was performed at laboratories of the College of Medicine, University of Malawi to amplify the LDH gene, a marker of *P. falciparum*, in the isolated total genomic DNA. The following published primer and probe sequences were used: Forward primer—ACG ATT TGG CTG GAG CAG AT; reverse primer—TCT CTA TTC CAT TCT TTG TCA CTC TTT C; probe—FAM-AGT AAT AGT AAC AGC TGG ATT TAC CAA GGC CCC A-TAMRA [38].

All reactions were run in duplicate on the StepOneplus Real-Time PCR machine (Applied Biosystems, Foster City, CA, USA). The cycling conditions were 50 °C for 2 min, 95 °C for 10 min, and 45 cycles of 95 °C for 15 s and then 60 °C for 1 min. Four *P. falciparum* 3D7 DNA serial dilutions (10, 1, 0.1, 0.01 ng/µl) were used as positive controls along with a non-template control. The threshold cycle (Ct) values were determined for each amplified duplicate after the threshold lines were manually placed for each reaction plate. Once both amplification curves crossed the threshold line, samples were deemed positive. Repeated reactions in the duplicate DNA template occurred when only one amplification curve crossed the threshold line, or the Ct value above 44.

### Anthropometric measurements

Anthropometric measurements were carried out by three trained research assistants during the enrolment visit. Unclothed infants were weighed using an electronic infant weighing scale (SECA 735; Chasmors Ltd. London, England) and weights were recorded to the nearest 10 g. The length was measured to the nearest 1 mm using a high-quality length board (Kiddimetre; Raven Equipment Ltd, Essex, England). Age- and sex-standardized anthropometric indices (length-for-age Z score [LAZ]), weight-for-age Z score [WAZ] and weight-for-length Z score [WLZ]) were calculated using the STATA macro developed by the World Health Organization (WHO) using the WHO 2006 multicentre growth standards [39].

### Statistical analysis

With the sample size of 210 participants allocated to each group, the original trial possessed an 85% likelihood of detecting significant contrasts in severe stunting rates. This was achieved while maintaining a two-sided Type I error of 5%, even with a potential 10% drop in participants due to attrition during the follow-up period. Additionally, the exclusion of individuals who were already severely stunted at the outset (as per the initial enrolment criteria) accounted for 5% of the sample. The proportion of positive malaria parasitaemia DBS samples by intervention group at enrollment, at 9, 12, 15, and 18 months was calculated separately, and for all intervention time points combined (9–18 months). All randomized participants were eligible to be included in the analyses, with the exception that subjects with missing data on an outcome variable were excluded from the analysis of that outcome. The modified Poisson regression with a robust variance estimator was used to estimate the prevalence ratio (PR) [40] between the control group and each three intervention groups at each time point separately, and for all time points combined. For all time points combined, the intragroup correlation due to multiple measurements per participant was taken into account by using robust standard errors for clustered data. The statistical analyses were conducted with Stata 17.0 (StataCorp, College Station, USA).

Null hypothesis of no difference between groups was rejected if P < 0.05. Wald’s test was used to test the global null hypothesis of no differences between the control group and the intervention groups, and the pairwise comparisons. For pairwise comparisons with P < 0.05, the hypothesis of no difference between groups was rejected only if the global null-hypothesis was also rejected.

The main analysis was conducted with covariate adjustments. Covariates used in the adjusted models were derived from a predefined list of variables that could modify the prevalence of malaria parasitaemia. The covariate selection with modified Poisson regression models was performed. All the models were adjusted for the same set of covariates, which were malaria parasitaemia at enrolment, season of DBS sample collection, study site, and household asset Z-score.

A sensitivity analysis was performed by combining children in the soy- and milk-LNS groups and compared it to the control group at any time of study visit to validate the association between LNS and the prevalence of asymptomatic malaria.

## Results

### Characteristics of the study participants

**Fig. 1:**
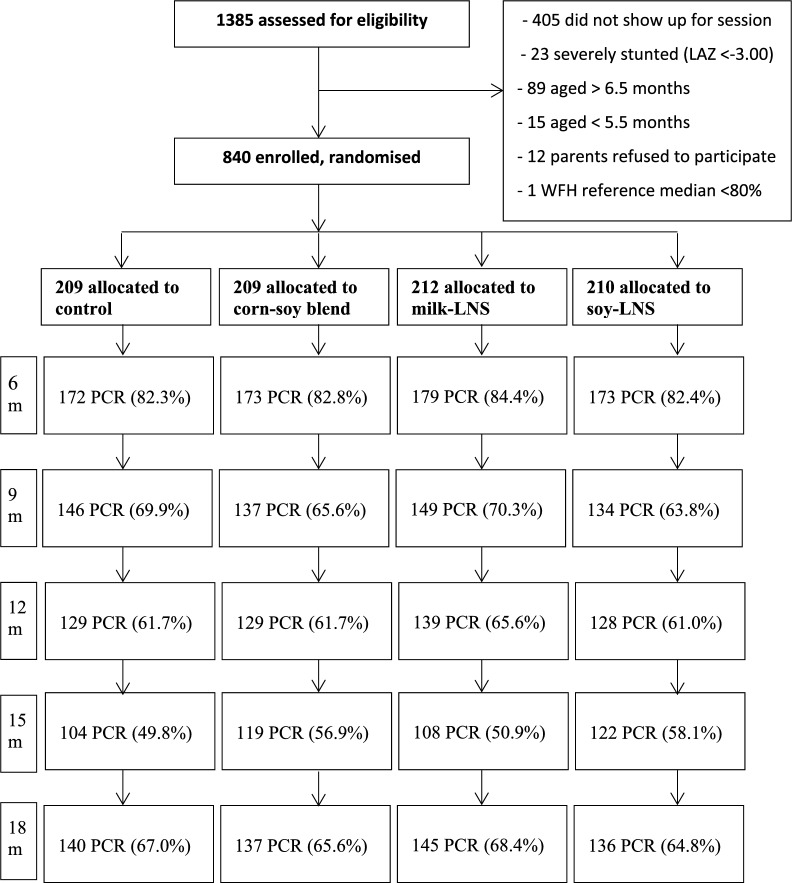
Flow diagram of participant data analyses. LNS = Lipid-based nutrient supplement; PCR = malaria results from PCR analysis of dried blood spots

A total of 1385 infants were screened, out of whom 840 were enrolled in the trial. The recruitment, group allocation, reasons for exclusions at enrollment, and success of their follow-up including available laboratory measurements at different visits are shown in Fig. 1. The participants were randomized into four groups: 209 to the control group, 209 to the CSB group, 212 to the milk-LNS group, and 210 to the soy-LNS group. Infants were not enrolled in the study because they were not brought to an enrollment session, they did not meet the inclusion criteria, or their guardians did not consent to participate in the study (Fig. 1). The proportion of excluded participants was similar in all groups at different visits.

There were no differences among intervention groups in terms of baseline characteristics (Table 2). The proportion of malaria prevalence at enrollment was 14.1%. Approximately 49% of the participating infants were boys. The mean (SD) weight and length were 7.0 (0.9) kg and 63.1 (2.3) cm, respectively. The mean LAZ, WAZ, WLZ were − 1.7 (1.0), − 0.8 (1.1) and 0.5 (1.0), respectively. The mean Hb concentration was 94.7 (16.8) g/L and mean maternal education was 3.5 (3.3) years. More than two thirds (73.2%) of the households used bed nets.

**Table 2:** Baseline characteristics of participants by intervention group

Participant characteristics	Intervention groups			
	Control	Corn-soy blend	Milk-LNS	Soy-LNS
Number of participants	209	209	212	210
Infant male sex, N (%)	109 (52.2%)	95 (45.5%)	105 (49.5%)	102 (48.6%)
Age (months)	6.02 (0.23)	6.03 (0.24)	6.02 (0.25)	6.04 (0.25)
Hb^a^ (g/L)	94 (17)	95 (16)	96 (17)	93 (17)
Weight (kg)	7.01 (0.89)	6.97 (0.99)	7.09 (0.97)	7.00 (0.88)
Length (cm)	63.1 (2.15)	62.9 (2.16)	63.2 (2.39)	63.0 (2.40)
WAZ	− 0.81 (1.05)	− 0.82 (1.17)	− 0.70 (1.11)	− 0.81 (1.12)
LAZ	− 1.66 (0.94)	− 1.70 (0.94)	− 1.59 (1.05)	− 1.69 (1.11)
WLZ	0.41 (1.05)	0.43 (1.09)	0.50 (1.05)	0.46 (1.01)
Household asset Z-score	0.02 (1.03)	0.00 (0.95)	0.01 (1.01)	− 0.03 (1.01)
Maternal education, years	3.51 (3.33)	3.69 (3.16)	3.81 (3.42)	3.04 (3.11)
Bed net use	145 (74.0%)	145 (72.5%)	146 (73.4%)	143 (73.0%)
Malaria prevalence, PCR	28 (16.3%)	20 (11.6%)	25 (14.0%)	25 (14.5%)

Data are presented as mean (± SD) unless otherwise stated

^a^Hemoglobin level < 110 g/L is considered anemic [55]

### Prevalence of asymptomatic malaria at different time points by intervention groups

Among all the collected blood samples, the proportion of positive malaria tests was 13.9%. After enrollment, the prevalence of asymptomatic malaria was 8.7% at 9 months, 11.2% at 12 months, 13.0% at 15 months, and 22.4% at 18 months of age.

The prevalence of asymptomatic malaria by visit and intervention group is shown in Table 3. There were statistically significant differences in the prevalence of malaria among intervention groups at all ages combined (P = 0.023) and at 15 months of age (P = 0.015). Compared to children in the control group and adjusting for potential confounders, the prevalence ratio of positive malaria test among children in the CSB group was 1.72 (95% CI 1.19–2.49; P = 0.004) at all ages combined and 3.24 (95% CI 1.46–7.17; P = 0.004) at 15 months of age. At 15 months of age, the prevalence ratio of positive malaria test among children in the soy-LNS group was 2.53, 95% CI 1.13–5.66; P = 0.024) compared to those in the control group.

**Table 3:** Prevalence of malaria at each study visit by the intervention group

Visit	The proportion of positive malaria tests by intervention group^a^					Comparison between control and intervention groups^b,c^					
						Control vs. CSB		Control vs. Milk-LNS		Control vs. Soy-LNS	
	Control	CSB	Milk-LNS	Soy-LNS	Global P-value	PR (95% CI)	P-value	PR (95% CI)	P-value	PR (95% CI)	P-value
All ages combined	12.1 % (63/519)	17.2 % (90/522)	12.2 % (66/541)	14.0 % (73/520)	0.023	1.72 (1.19, 2.49)	0.004	1.19 (0.81, 1.74)	0.372	1.32 (0.88, 1.96)	0.177
9 months	6.8 % (10/146)	12.4 % (17/137)	8.1 % (12/149)	7.5 % (10/134)	0.088	2.63 (1.20 to 5.75)	0.015	1.60 (0.68 to 3.76)	0.286	1.66 (0.68 to 4.06)	0.267
12 months	8.5 % (11/129)	15.5 % (20/129)	10.1 % (14/139)	10.9 % (14/128)	0.329	1.86 (0.92 to 3.76)	0.083	1.29 (0.59 to 2.82)	0.531	1.33 (0.62 to 2.90)	0.465
15 months	8.7 % (9/104)	18.5 % (22/119)	8.3 % (9/108)	15.6 % (19/122)	0.015	3.24 (1.46 to 7.17)	0.004	1.50 (0.57 to 3.90)	0.411	2.53 (1.13 to 5.66)	0.024
18 months	23.6 % (33/140)	22.6 % (31/137)	21.4 % (31/145)	22.1 % (30/136)	0.957	1.02 (0.64 to 1.61)	0.936	1.00 (0.63 to 1.60)	0.988	0.90 (0.56 to 1.45)	0.671

PR: Prevalence ratio; CI: Confidence interval; CSB: Corn-soy blend; Milk-LNS: Milk-powder containing lipid-based nutrient supplement; Soy LNS: Soy flour containing lipid-based nutrient supplement

^a^Unadjusted proportion of malaria prevalence

^b^Prevalence ratio, obtained using a modified Poisson regression (with a robust variance estimator). The clustering of participants was taken into account for combined time points

^c^Adjusted for malaria at baseline, season of sample collection, site of enrolment, and household asset Z-score

A sensitivity analysis combining children from the soy-LNS and milk-LNS groups into one group confirmed that there was no association between LNS provision and asymptomatic malaria at any of the study visits (see Additional file 1: Appendix Table S1).

## Discussion

The present study evaluated the impact of three different dietary interventions on the occurrence of asymptomatic malaria among 6–18 months old Malawian children. This study reported the overall prevalence of asymptomatic malaria among young children as 14.1% in rural Malawi. There was no statistically significant association found between LNS provision and the prevalence of asymptomatic malaria. In contrary to the original hypotheses, the provision of CSB was associated with a higher malaria prevalence.

Randomized study design, large sample size, investigators blinded to the samples’ status, and use of real-time PCR (sensitive and accurate method for detecting asymptomatic malaria cases) reduced the probability of random error or bias in the current study. Differences in laboratory methods, excluded participants or those with missing data (due to either loss of cases, visits, or blood specimens) could have affected internal validity. Nevertheless, standard procedures for laboratory sample collection, storage, and methods were used across the study. Furthermore, included and excluded participants had similar baseline characteristics. Therefore, the findings of this study are valid and representative and not supportive of a hypothesis that the tested dietary supplements would reduce the prevalence of asymptomatic malaria prevalence in this target population of 6–18-month-old children in Malawi.

There are a limited number of clinical trials that have evaluated the potential effects of micronutrient supplements on children living in malaria-endemic areas which lead to different outcomes either protective, adverse, or no effects [31, 41]. In this study, CSB and LNS interventions have not decreased the prevalence of asymptomatic malaria compared to the control group which did not support the study hypothesis. These might be more related to nutrient composition and the infant’s immune response to asymptomatic malaria. Some of the micronutrient components that were also included in the study daily intervention supplements were proven in previous studies to have a protective impact on the decreased prevalence of malaria including zinc [42] and no effect was found with thiamine [43] as a source of nutrients and energy. While iron [44, 45] and folate [46] have been found to exacerbate malaria morbidity, and riboflavin had an unclear impact [47].

In this study, CSB supplementation was associated with a higher malaria prevalence among 6–18-month-old children. Among the three intervention used in this study, CSB has a lower nutrient density and it was associated with relatively poor linear growth outcomes in a prior study [36]. Giving a diluted voluminous porridge mixture of CSB to infants might reduce or displace normal breastfeeding and any other home dietary food intake. This in turn might affect innate and adaptive children´s immunity and increase susceptibility to malaria infection [48, 49]. Compared to LNS, CSB supplement has a lower content of retinol (Vitamin A) and zinc with no vitamin D, all of which are considered essential for the normal immune function to modulate malaria morbidity and mortality in different aspects [31, 33, 50, 51]. CSB also lacked the pantothenic acid (vitamin B5) and selenium trace element that both were found in previous studies to have anti-plasmodial activity against *P. falciparum* [52–54]. These explanations are the possible assumptions that need more proof and clarification otherwise considered random findings. Therefore, more research is needed to understand better the exact mechanism for the complex interaction of micronutrients on immunity and malaria.

## Conclusion

The study findings did not support the hypothesis that children between 6 to 18 months who receive daily dietary supplementation with LNS or CSB would have a lower risk of asymptomatic malaria in rural Malawi. In contrast, there was a signal of a possible increase in malaria prevalence among children supplemented with CSB. It would be important to monitor malaria parasitaemia with adequate control measures when further studies will be done with micronutrients fortified CSB.

### Supplementary Information

**Additional file 1: Table S1.** Prevalence of malaria at each study visit by the intervention group (sensitivity analysis).

## Data Availability

Data described in the manuscript, codebook, and analytic code will be made available upon request pending approval by the authors.

## Abbreviations

LNS: Lipid-based nutrient supplements
CSB: Corn-soy blend
DBS: Dry blood spot
LCNI-5: Lungwena Child Nutrition Intervention 5
PCR: Polymerase chain reaction
PBS: Phosphate-buffered saline
LDH: Lactate dehydrogenase gene
LAZ: Length-for-age Z score
WAZ: Weight-for-age Z score
WLZ: Weight-for-length Z score
SD: Standard deviation
WHO: World Health Organization
RR: Risk ratio
CI: Confidence interval
IgG: Immunoglobulin G
