# Supplementary material for: Effect of dietary intervention on the prevalence of asymptomatic malaria among 6–18-month-old children in rural Malawi
Source: Malar J. 2023 Sep 11;22:266. doi: 10.1186/s12936-023-04701-4 (PMC10496296; doi:10.1186/s12936-023-04701-4)
Supplement: Supplementary file 1 — Additional file 1: Table S1. Prevalence of malaria at each study visit by the intervention group (sensitivity analysis). [file 12936_2023_4701_MOESM1_ESM.docx]

**Table S1.** **Prevalence of malaria at each study visit by the intervention group (sensitivity analysis)**

|  | **The proportion of positive malaria tests by intervention group^a^** | | | | **Comparison between control and intervention groups^b,c^** | | | |
| --- | --- | --- | --- | --- | --- | --- | --- | --- |
| **Visits** | **Control** | **CSB** | **Milk/Soy-LNS** | **Global**  **P-value** | **Control vs. CSB** | | **Control vs. Milk/Soy-LNS** | |
|  |  |  |  |  | **PR (95% CI)** | **P-value** | **PR (95% CI)** | **P-value** |
| **All ages combined** | 12.1 % (63/519) | 17.2 % (90/522) | 13.1 % (139/1061) | 0.010 | 1.72  (1.19, 2.49) | 0.004 | 1.25  (0.89, 1.77) | 0.204 |
| **9 months** | 6.8 % (10/146) | 12.4 % (17/137) | 7.8 %  (22/283) | 0.039 | 2.63  (1.20, 5.75) | 0.015 | 1.63  (0.75, 3.53) | 0.219 |
| **12 months** | 8.5 % (11/129) | 15.5 % (20/129) | 10.5 % (28/267) | 0.180 | 1.86  (0.92, 3.76) | 0.083 | 1.31  (0.66, 2.62) | 0.445 |
| **15 months** | 8.7 %  (9/104) | 18.5 %  (22/119) | 12.2 % (28/230) | 0.011 | 3.25  (1.46, 7.20) | 0.004 | 2.01  (0.91, 4.43) | 0.082 |
| **18 months** | 23.6 % (33/140) | 22.6 % (31/137) | 21.7 % (61/281) | 0.939 | 1.02  (0.64, 1.61) | 0.936 | 0.95  (0.64, 1.43) | 0.816 |

^a^ Unadjusted proportion of malaria prevalence.

^b^Prevalence ratio, obtained using a modified Poisson regression (with a robust variance estimator). We took the clustering of participants into account for combined time points.

^c^Adjusted for malaria at baseline, season of sample collection, site of enrolment, and household asset Z-score.

PR = Prevalence ratio; CI = Confidence interval; CSB = Corn-soy blend; Milk-LNS = Milk-powder containing lipid-based nutrient supplement; Soy LNS = Soy flour containing lipid-based nutrient supplement
